# Dynamic mechanisms and spatial spillover effects of cultural tourism development in the China–Pakistan Economic Corridor

**DOI:** 10.3389/fpsyg.2025.1555524

**Published:** 2025-06-16

**Authors:** Zuhaib, Gang Liu, Jingyao Chen, Shuaib Ali, Zhang Rui, Dawood, Huan Chen

**Affiliations:** ^1^College of International Tourism and Public Administration, Hainan University, Haikou, China; ^2^School of Business, Macau University of Science and Technology, Taipa, Macao SAR, China; ^3^School of Management, Guangzhou University, Guangzhou, China; ^4^Qiongtai Teachers College, Qiongtai Normal University, Hainan, China

**Keywords:** cultural tourism, Hofstede’s cultural dimensions theory, spatial spillover effects theory, dynamic mechanism, regional development, SDM, CPEC

## Abstract

**Introduction:**

This study explores the spatial spillover effects (SSE) and dynamic mechanisms of cultural tourism in the China–Pakistan Economic Corridor (CPEC). This study examines how infrastructure development, cultural integration, and spatial connectivity affect cultural tourism in Pakistan’s CPEC and non-CPEC regions using spatial spillover and Hofstede’s cultural dimensions theory.

**Methods:**

Data were collected from 1,387 respondents in both regions, analyzed using the spatial Durbin model and structural equation modeling, and visualized using ArcMap and Geoda.

**Results:**

The results indicate that the growth of cultural tourism is strongly influenced by dynamic processes, including better infrastructure, more cultural interchange, and easier accessibility, particularly in CPEC regions. Furthermore, non-CPEC regions exhibit minor advantages in terms of indirect economic gains and increased accessibility, highlighting the ongoing inequalities.

**Discussion:**

These findings provide empirical insights into the regional impacts of large-scale initiatives, such as CPEC, and draw attention to government intervention, equitable resource allocation, and stakeholder collaboration to achieve long-term cultural tourism.

## 1 Introduction

The China–Pakistan Economic Corridor (CPEC) was formally launched in 2015 as a key component of China’s Belt and Road Initiative (BRI), aiming to enhance regional trade and economic ties between Pakistan and China (Nazneen et al., 2019). With over $60 billion in investment in trade, energy, and infrastructure development, CPEC strengthens bilateral ties and holds significant potential for boosting cultural tourism (Asif and Ling, 2018). Cultural tourism, which involves engagement with historical landmarks, traditional arts, and local practices, has emerged as a dynamic sector that fosters economic revitalization, heritage preservation, and social cohesion (Chen and Rahman, 2018; Richards, 2018). The benefits of cultural tourism are well documented in the literature, ranging from economic revitalization (Torre and Scarborough, 2017) and historic preservation (Chen and Rahman, 2018) to social capital development (Martínez-Pérez et al., 2019; Zheng et al., 2023). It also supports sustainable development and empowers communities to safeguard their cultural heritage while contributing to economic resilience and social equity (Wu et al., 2020).

Within the CPEC framework, cultural tourism presents a significant opportunity to influence the region’s rich cultural diversity and heritage to attract both domestic and international tourists (Conrad, 2017; Hameed et al., 2022). However, despite its potential, the dynamic mechanisms and spatial spillover effects (SSE) of cultural tourism within the CPEC initiative remain largely unexplored. Existing literature has primarily focused on the socioeconomic benefits and designs of cultural tourism (Torre and Scarborough, 2017; Wu et al., 2020) or the developmental implications of infrastructure projects (Nazneen et al., 2019; Kumar and Dhir, 2020). While these studies provide valuable insights, limited attention has been paid to understanding how cultural values shape traveler behavior or how major infrastructure projects, such as CPEC, influence the spatial distribution of tourism-related benefits. Despite the significance of SSE, there remains a research gap in evaluating how large-scale initiatives like CPEC influence cultural tourism across spatially linked regions. To the best of the authors’ knowledge, this study is among the first to empirically examine the spillover effects of CPEC investments on tourism dynamics and cultural preservation. This study fills this crucial gap by integrating Hofstede’s principle of cultural dimensions with the theory of spatial spillover. This research investigates the diffusion of benefits across 64 districts in CPEC and non-CPEC regions, providing a comprehensive understanding of the interconnected impacts. This study has two main objectives: (i) to examine how residents’ and visitors’ behaviors, perceptions, and socioeconomic effects of cultural tourism are influenced by Hofstede’s first five cultural dimensions in both CPEC and non-CPEC regions. (ii) To investigate the SSE of cultural tourism within the CPEC framework by assessing how infrastructure investments produce cultural and economic benefits in neighboring regions. This study makes several contributions to the literature. First, it is the first study to apply both SST and Hofstede’s cultural dimensions to large-scale infrastructure projects such as CPEC. Second, it provides empirical evidence on how cultural values and infrastructure investments interact, shaping local tourism patterns and socioeconomic outcomes. Third, it explores dynamic mechanisms and evaluates the SSE of cultural tourism, providing practical suggestions for strengthening equitable and sustainable regional development. Finally, this study provides actionable recommendations for policymakers, illustrating how CPEC can serve as a model for cultural preservation, inter-regional cooperation, and infrastructure-driven tourism growth, ultimately fostering long-term socioeconomic resilience.

The structure of this study is as follows: Section 2 discusses the theoretical background, including a review of existing research and an integration of spatial spillover and Hofstede’s theory. Section 3 outlines the research methodology, detailing data collection and analysis techniques. Section 4 presents the results and key findings, followed by Section 5, which discusses theoretical and practical implications. Finally, Section 6 highlights the study’s limitations and suggests directions for future research.

## 2 Theoretical background and hypotheses development

### 2.1 CPEC initiatives and cultural tourism

The CPEC is a flagship initiative of China’s BRI that aims to advance economic growth and regional integration through trade via large-scale infrastructure investments. It encompasses a 3,218-kilometer network of highways, railways, pipelines, and economic zones that link Gwadar Port (Pakistan) to Xinjiang Province (China). Strategically, it provides China with direct access to the Arabian Sea, enhancing maritime connectivity and transforming regional shipping dynamics (Siddiqi, 2018). Several scholars have highlighted the broader geopolitical and maritime significance of the CPEC. For instance, CPEC has enhanced maritime security collaboration between China and Pakistan, underlining its dual role as both an economic and strategic initiative (Chang and Khan, 2019). As part of the 21st Century Maritime Silk Road, the CPEC contributes to China’s naval diplomacy and port development strategy in the Indian Ocean, influencing regional maritime governance (Chang, 2018; Chang and Khan, 2019). These developments echo the shifts observed in global port cooperation frameworks, as seen in comparative contexts such as post-Brexit maritime trade realignments (Khan et al., 2022). The CPEC project exceeds an investment of $60 billion, including $18.1 billion from domestic sources, to modernize infrastructure, overcome energy shortages, and attract foreign investment through economic zones (Hussain, 2019; Kumar and Dhir, 2020; Hameed et al., 2022). Beyond its trade and infrastructure goals, CPEC presents significant opportunities for enhancing cultural tourism in the underdeveloped regions of Pakistan, leveraging improved connectivity and investment inflows. Cultural tourism honors the spiritual, intellectual, and emotional legacies of community identity (Richards, 2018). Similarly, cultural tourism generates socioeconomic benefits such as strengthening community identity, facilitating intercultural communications (Richards, 2018; Martínez-Pérez et al., 2019; Zheng et al., 2023), preserving cultural heritage (Wu et al., 2020), establishing community engagement, generating economic opportunities, and promoting cross-cultural interactions (Torre and Scarborough, 2017; Nazneen et al., 2019).

CPEC’s infrastructure advancements, such as improved transportation networks and enhanced energy access, create opportunities to make cultural tourism more accessible and attractive. These developments can transform tourism into a key driver of economic growth, particularly in rural and culturally rich regions. For example, upgraded roadways under the CPEC enhance access to heritage sites, fostering regional development and cross-border collaboration (Nazarian et al., 2017; Mian, 2021; Hameed et al., 2022). Effective governance and equitable resource distribution are critical for ensuring that CPEC’s benefits extend beyond economic gains and include cultural preservation, social well-being, and sustainable tourism practices.

### 2.2 Hofstede’s cultural theory and cultural tourism

Understanding cultural influences on tourism requires a multi-dimensional approach. Several cultural frameworks have been developed to analyze how behaviors and values shape social and economic interactions. Hall’s (1976) cultural theory distinguishes between high- and low-context cultures, offering insights into how communication styles affect tourist experiences and intercultural engagement (Hall, 1976). A recent study applied Hall’s framework in tourism to emphasize how misaligned communication expectations can result in visitor dissatisfaction and misunderstandings (Chen et al., 2023). Similarly, Trornpenaars and Hampden-Turner (1998) expanded on this by proposing seven cultural dimensions, including universalism vs. particularism and achievement vs. ascription, which help explain cross-cultural differences in perception, behavior, and travel expectations (Smith et al., 1996). A recent study highlighted how Trompenaars’ dimensions inform tourism behavior across regions, making them relevant for global tourism management (Trompenaars and Hampden-Turner, 2011). Additionally, Hofstede’s cultural theory offers a structured framework for understanding how cultural values influence social behaviors, interpersonal interactions, and decision-making (Hofstede, 2001). While all these models provide valuable perspectives, Hofstede’s cultural dimensions theory remains the most widely applied in tourism studies because of its empirical depth and clarity. Hofstede’s cultural dimensions theory was initially established by Geert Hofstede in the 1970s and has since been modified over the decades (Hofstede, 2001).

This theoretical framework is widely used in tourism research to examine how cultural factors impact tourist choices, travel behavior, and satisfaction (Reisinger and Crotts, 2010; Annamoradnejad et al., 2019). The first five Hofstede dimensions, namely the Power Distance Index (PDI), Uncertainty Avoidance Index (UAI), Individualism vs. Collectivism (IDV), Masculinity vs. Femininity (MAS), and Long-term vs. Short-term Orientation (LTO), provide a useful framework for examining how cultural values impact the behaviors, attitudes, and socioeconomic effects of cultural tourism among locals and visitors (Hofstede, 2001; Kirkman et al., 2006; Hofstede, 2011; Litvin, 2019; Mele et al., 2021). The PDI measures the extent to which a society accepts unequal power distribution. Numerous studies have revealed mixed findings regarding its impact on tourism. For example, Huang and Crotts (2019) and Mele et al. (2021) found that a high PDI society negatively impacts tourist satisfaction, whereas Correia et al. (2011) and Kumar and Dhir (2020) indicated that the PDI influence on tourism is statistically insignificant. On the other hand, Gao et al. (2018) and Pagda et al. (2021) suggested that high-PDI societies tend to prioritize decision-making, potentially restricting the inclusion of tourist strategies.

CPEC’s emphasis on decentralization may mitigate these effects by promoting more inclusive tourism policies. UAI reflects a society’s desire for structure and predictability. Research has highlighted that UAI has a significant impact on tourism dynamics. It was found that higher UAI is correlated with higher tourist spending (Gholipour et al., 2014) and has a positive impact on travel choices and post-trip satisfaction (Litvin, 2019; Kanwal et al., 2020). Similarly, a lower UAI attracts more tourists and has diverse impacts on society (Zhang et al., 2019). IDV highlights the contrast between individualistic and collectivist cultures, especially in tourism, where its correlation varies. Individualistic cultures place strong emphasis on individual liberty and autonomy, which fuels tourism competition and shapes travel interests (Mele et al., 2021; Pagda et al., 2021). In the meantime, collectivist cultures encourage social harmony and often attract more tourists (Liao, 2016; Lim and Ok, 2021). Tourist satisfaction is positively correlated with high IDV (Huang and Crotts, 2019; Kumar and Dhir, 2020). CPEC initiatives could enhance the preservation of collectivism while promoting individualistic societies (IDV) through cultural interactions and travel choices. The MAS dimension addresses gender roles and priorities in society. Sustainable tourism is more appropriate in feminine societies, which are classified by teamwork and community identity (Nazarian et al., 2017; Bogatyreva et al., 2019).

However, Kumar and Dhir (2020) claimed that economic competitiveness is highly appreciated in masculine societies. CPEC has the potential to mitigate these trends and may make it possible for more inclusive and community-driven tourism practices. LTO and STO are measures of how a culture perceives benefits in the future compared to present achievements. It has been shown a negative relationship between STO and LTO on immediate tourism outcomes (Gholipour et al., 2014; Kumar and Dhir, 2020). The STO has a positive impact on tourist arrivals, whereas high-LTO attracts more tourists owing to significant investments in infrastructure development (Huang and Crotts, 2019; Mele et al., 2021). It is expected that CPEC’s focus on sustainability and global best practices will improve LTO while mitigating STO traits. Based on existing research, this study combines Hofstede’s dimensions with the CPEC’s cultural tourism infrastructure to formulate the following hypotheses:

*H1:* CPEC initiatives will positively reduce PDI by promoting decentralization and inclusive leadership.

*H2:* CPEC initiatives will significantly reduce UAI by improving infrastructure reliability and connectivity.

*H3:* CPEC initiatives will positively increase IDV by fostering diverse travel opportunities and cultural interactions.

*H4:* CPEC initiatives will significantly balance MAS by supporting sustainable and community-based tourism activities.

*H5:* CPEC initiatives will positively improve LTO and reduce STO by advancing stable tourism and long-term infrastructure projects.

### 2.3 Spatial spillover effects of cultural tourism

Spatial Spillover Effects (SSE) refer to the phenomenon where economic, cultural, and social changes initiated in one region influence surrounding regions, either positively or negatively. In the context of cultural tourism, SSE describes how investments in infrastructure, tourism services, and cultural preservation in one district can indirectly promote tourism activity, economic resilience, and regional collaboration in neighboring districts. For instance, a new cultural site developed under CPEC projects may increase tourist flow not only to that location but also to nearby areas through improved accessibility, enhanced visibility, and shared cultural heritage (Capello, 2009; Zhou et al., 2022). The spatial spillover theory (SST) provides a conceptual foundation for understanding this dynamic by connecting localized development with regional transformation. SST suggests that strategic investments, particularly those related to connectivity and culture, generate cumulative benefits, especially when spatial proximity and socioeconomic linkages are strong (LeSage and Pace, 2009; Shi et al., 2021; Li et al., 2022; Zhou et al., 2022). SST also investigates how local investments generate regional benefits beyond direct borders, creating interconnected economic and cultural cooperation (Capello, 2009). Interconnected tourism investments share economic benefits and stimulate regional collaboration (Zheng et al., 2023). Similarly, the role of coordinated investments in promoting sustainable tourism development across regions (Fan and Xue, 2020) and tourism-related investments boosts visitation in neighboring regions through improved accessibility and shared cultural assets (Li et al., 2022; Liao and Liang, 2024). In the context of the CPEC project, SST implies that cultural tourism enhancements in one district may “spill over” to adjacent non-CPEC regions, thereby fostering more inclusive and sustainable development across borders. Improved infrastructure and regional connectivity under CPEC infrastructure development, such as the construction of roads, railways, and special economic zones, are expected to act as catalysts for regional collaboration and cultural tourism development. These spillovers can potentially lead to enhanced tourism activities, economic development, and cultural heritage preservation across neighboring areas, aligning with the broader objectives of sustainable tourism.

## 3 Methodology

### 3.1 Sampling strategy and respondent selection

This study employed a stratified sampling technique to ensure representativeness across 64 districts in Pakistan, including 32 in CPEC regions and 32 in non-CPEC regions. The respondents included a mix of students, government and private sector employees, entrepreneurs, tourism workers, and other local stakeholders engaged in or affected by cultural tourism and regional development. They were selected based on geographic location and demographic factors, such as age, gender, education, and occupation. This approach mitigates self-selection bias and ensures diversity from the cultural tourism perspective. A pilot study was conducted with 50 graduate students from the University of Swat to assess the reliability and clarity of the questionnaire. The results showed high internal consistency with a Cronbach’s alpha of 0.874. After the final deployment, 1,561 responses were received, of which 1,387 responses (719 from CPEC and 668 from non-CPEC regions) were complete and deemed valid for analysis.

### 3.2 Data collection procedures

This study utilized a quantitative survey method administered via multiple channels to enhance reach and accessibility. The survey was initially distributed using Google Forms and later expanded to include social media platforms (e.g., Facebook groups, WhatsApp communities), email invitations, and offline networks to increase inclusiveness and participation. The questionnaire, originally designed in English, was translated into Urdu and other regional languages to accommodate respondents from diverse linguistic backgrounds and improve their understanding. To further improve data quality, multi-modal collection and response validation were employed. The combination of diverse distribution methods, stratified sampling, and response validation measures reinforces the methodological rigor of this study, ensuring both the reliability and representativeness of the findings.

### 3.3 Measurement items

All items were assessed using multiple-item measures and refined to fit the context of this study, as shown in Appendix. The first part of the questionnaire collected respondents’ demographic details (age, gender, education level, and type of profession), while the second part measured the impact of CPEC infrastructural development with five items adopted from previous studies (Hussain, 2019; Zulfaqar et al., 2023). The third part of the study examined Hofstede’s cultural dimensions, with each assessed using five validated items adapted from previous research. The power distance index (PDI) was measured using established scales from prior studies (Dorfman and Howell, 1988; Hofstede, 2001; Nazarian et al., 2017; Filimonau et al., 2018). Similarly, the uncertainty avoidance index (UAI) was evaluated using a consistent framework (Dorfman and Howell, 1988; Hofstede, 2001; Filimonau et al., 2018). The selected items for individualism vs. collectivism (IDV) were sourced from widely accepted cultural research (Hofstede, 2001; Nazarian et al., 2017). The masculinity vs. femininity (MAS) dimension was assessed using validated indicators to capture gender-related cultural distinctions (Dorfman and Howell, 1988; Nazarian et al., 2017; Filimonau et al., 2018). Finally, long-term vs. short-term orientation (L/STO) was measured using rigorously tested items from previous studies (Nazarian et al., 2017; Filimonau et al., 2018).

### 3.4 Econometric model

#### 3.4.1 Spatial data analysis (SDA)

To operationalize spatial spillover effects (SSE), this study uses spatial autocorrelation and econometric techniques that allow us to statistically measure how tourism development in one district influences others. Spatial autocorrelation is a fundamental aspect of SDA within Geographic Information Systems (GIS), a statistical method used to reveal the spatial distribution patterns and relationships of geographic phenomena (Anselin, 1995). Spatial autocorrelation can be categorized into two types: global and local. Global spatial autocorrelation, often measured using Moran’s I, is particularly effective in assessing spatial correlation, dependence, and interactions among spatial variables. The equation for global Moran’s I is as follows (Anselin, 1995):


(1) $$I=\sum_{i=1}^{n}\sum_{j\neq i}^{n}w_{ij}\left(x_{i}-xˉ\right)\left(y_{j}-yˉ\right)/S^{2}\sum_{i=1}^{n}\sum_{j\neq i}^{n}w_{ij}.$$

In Equation 1, “I” represents the global indicator for measuring global spatial autocorrelation, where a higher “I” value indicates stronger spatial clustering between CPEC and non-CPEC regions. Variables xi and yi denote the observed values for districts i and j, respectively, and *n* is the total number of districts. Similarly, S^2^ represents the sample variance, and wij is the spatial weight matrix constructed based on neighborhood criteria, with wij = 1 for neighboring districts and wij = 0 otherwise. This equation captures the spatial relationships between variables across spatial units, accounting for spillover effects through the spatial weight matrix. Local Indicators of Spatial Association (LISA) were used to identify specific patterns.

The Local Moran’s I equation is as follows:


(2) $$I_{i}=\frac{\left(y_{j}-yˉ\;\right)}{s^{2}}\sum_{i=1,j\neq i}^{n}w_{ij}\left(y_{j}-yˉ\;\right)$$

In Equation 2, “Ii” represents the local spatial correlation levels between the CPEC regions in district i and the non-CPEC regions in district j. Local Moran’s I can be classified into four distinct categories: low–low (LL), high–high (HH), low–high (LH), and high–low (HL). LL and HH indicate that the CPEC region in district i is significantly spatially correlated with the non-CPEC regions in district j, where districts with high (or low) economic activity are surrounded by districts with similarly high (or low) levels. Conversely, HL and LH reflect a negative spatial correlation between the concentrations of CPEC regions in district i and non-CPEC regions in district j. Additionally, in the case of a significant local Moran’s I result, we further applied multi-variable Local Spatial Autocorrelation Analysis (LISA) to map spatial clusters and hotspots using ArcMap and GeoDa, ensuring a more comprehensive spatial interpretation.

#### 3.4.2 Spatial econometric model

Traditional econometric models based on linear correlations frequently fail to capture the spatial dependencies and geographical spillover effects (Cliff et al., 1981). Once spatial dependence is achieved through spatial autocorrelation analysis, it is crucial to construct a spatial econometric model to examine regional interactions (Elhorst, 2003; Xu et al., 2020). Spatial econometric models commonly used for such purposes, including the spatial error model (SEM), spatial lag model (SLM), and spatial Durbin model (SDM), are preferred because they account for spatial influences on both dependent and independent variables, offering greater scope and expanded explanatory power compared to SEM and SLM. Hence, we apply the Spatial Durbin Model (SDM), which is particularly suitable for capturing both direct effects (e.g., how local cultural dimensions influence tourism within the same district) and indirect or spillover effects (e.g., how those same dimensions impact neighboring districts through spatial interaction) across 64 districts in Pakistan. The general form of the SDM denoted by Equation 3 as follows:


(3) y=ρWy+Xβ+WX θ+∈

Detailed equation:


(4) $$\begin{array}{c} \begin{array}{l} yi=\rho \sum_{j=1}^{n}w_{ij}y_{j}+\beta_{1}{\text{PDI}}_{i}+\beta_{2}IDV_{i}+\beta_{3}MAS_{i}+\\ \beta_{4}UAI_{i}+\beta_{5}LSTO_{i}+\theta_{1}\sum_{j=1}^{n}w_{ij}PDI_{j}+ \end{array}\\ \begin{array}{l} \theta_{2}\sum_{j=1}^{n}w_{ij}IDV_{j}+\theta_{3}\sum_{j=1}^{n}w_{ij}MAS_{j}+\\ \theta_{4}\sum_{j=1}^{n}w_{ij}UAI_{j}+\theta_{5}\sum_{j=1}^{n}w_{ij}LSTO_{j}+\in_{i} \end{array} \end{array}$$

In the above Equation 4, *yi* represents the concentration value of the dependent variable for district *i* in either the CPEC regions or the non-CPEC region. The spatial weight matrix is represented by wij based on the neighborhood relationship between district *i* and district *j*, including the spatial relationships between all districts in the dataset, covering both CPEC regions and non-CPEC regions, as in the above-drawn Equation 1. The spatial autoregressive coefficient for the dependent variable is denoted by the term ρ (rho), which measures the strength of the SSE in the context of the dependent variable (tourism performance) across districts from both regions. A positive ρ reflects that improvements in one district are associated with better outcomes in neighboring districts, confirming the presence of positive spatial spillovers. Conversely, a negative ρ might suggest competition or resource cannibalization between districts. The vectors β and θ represent the coefficients related to the direct impact of the independent variables (PDI, IDV, MAS, UAI, and L/STO) and their spatially lagged counterparts, respectively. Specifically, *β*1, *β*2, *β*3, *β*4, and *β*5 are the coefficients of parameter vectors, and *θ*1, *θ*2, *θ*3, *θ*4, and *θ*5 are the coefficients for the spatially lagged terms of PDI, IDV, MAS, UAI, and LTO, respectively. The term *ε* is the vector of error terms, capturing the unobserved factors affecting the dependent variable. By analyzing the SSE in these ways, the model not only evaluates localized cultural tourism performance but also indicates broader regional dynamics essential for cross-border development and collaborative policy-making within and beyond CPEC corridors.

### 3.5 Data analysis techniques

As shown in Table 1, descriptive statistics of the demographic data were analyzed using RStudio. The sample comprised 907 males (65.39%) and 480 females (34.61%). The age groups were distributed as follows: 88 aged 18, 324 aged 19–28, 602 aged 29–40, 278 aged 41–50, and 95 aged 51 years and above. In terms of education, 783 respondents held a bachelor’s degree or below, 400 held a master’s degree, and 204 held a PhD. Professionally, 259 respondents were students, 175 were government employees, 221 were private sector employees, 213 were self-employed or entrepreneurs, 240 worked in the tourism sector, and 279 were engaged in other professions. These demographics help contextualize how different social groups perceive cultural tourism development across CPEC and non-CPEC regions. Common Method Bias (CMB) was applied in RStudio using Harman’s single-factor test to assess CMB in the datasets. Then the PLS-SEM (Partial Least Squares Structural Equation Modeling) method was used for hypothesis testing and analyzing the relationships among Hofstede’s cultural dimensions (PDI, UAI, IDV, MAS, and L/STO). The model was tested using Smart-PLS version 4.1, and the reliability and validity of the constructs were confirmed through composite reliability, AVE, and outer loading VIF. Similarly, spatial data analysis was conducted to explore the geographic clustering of cultural tourism’s impacts. The techniques included Global Moran’s I and Local Indicators of Spatial Association (LISA). Additionally, we applied the Spatial Durbin Model (SDM) to assess the spatial spillover effects (SSE) of cultural and infrastructural variables between the CPEC and non-CPEC regions.

**Table 1:** Participants profile.

Participants description	CPEC regions		Non-CPEC regions	
	Frequency	Percentage	Frequency	Percentage
Gender				
Male	406	56.46	417	62.42
Female	313	43.53	251	37.57
Age				
18 years	43	05.98	45	6.73
19–28 years	168	23.36	156	23.35
29–40 years	310	43.11	292	43.71
41–50 years	144	20.05	134	20.05
Above 51 years	54	7.510	41	6.130
Qualification				
Matric	103	14.32	107	14.67
Intermediate	127	17.66	114	17.36
Bachelors	179	24.89	161	24.25
Masters/M. Phil	209	29.06	193	28.29
PhD	101	14.05	93	13.92
Profession				
Student	138	19.19	121	18.11
Government employee	93	12.93	82	12.27
Private employee	98	13.63	123	18.41
Entrepreneur/self-employed	126	17.52	87	13.02
Tourism workers	161	22.39	79	11.82
Other	103	14.32	176	26.34

Source: created by the author from survey data using smart-PLS.

## 4 Results

### 4.1 Common Method Bias (CMB)

As the data in this study were gathered from a single source, we took proactive steps to address potential CMB using both procedural and statistical methods (Podsakoff et al., 2012). To mitigate CMB during data collection, we implemented the preemptive strategies outlined by Spector (2006), including maintaining respondent anonymity and confidentiality to minimize response biases. To statistically assess CMB, we applied two established methods and confirmed the normality of the data using skewness and kurtosis tests. First, we conducted Harman’s single-factor test through exploratory factor analysis (EFA), which is a widely accepted method for testing CMB, regardless of data normality. Second, we employed the full collinearity variance inflation factor (VIF) method suggested by Kock (2015). The results of the first factor explained 26.80% of the total variance, which was well below the 50% threshold typically associated with severe CMB (Harman, 1976; Podsakoff et al., 2012), whereas the VIF values ranged from 1.07 to 3.23, confirming that CMB was not a significant issue in this study.

### 4.2 Measurement model

The reliability and validity of the PDI, UAI, IDV, MAS, and LSTO measures were assessed using PLS, as shown in Table 2. Internal consistency was confirmed with cross-loadings exceeding the cut-off value (>0.5), Cronbach’s alpha ranging from 0.752 to 0.895, composite reliability ranging from 0.841 to 0.923, and average variance extracted (AVE) from 0.558 to 0.706, confirming reliability and convergent validity (Fornell and Larcker, 1981). Discriminant validity was confirmed using the Heterotrait-Monotrait (HTMT) ratio, with all values below 0.85, as shown in Table 3 (Voorhees et al., 2016). A normality test, essential for SEM, was conducted using the R software (Cain et al., 2017). Table 3 presents the skewness and kurtosis values for all variables, which met the criteria for normality, with skewness between ±1.5 and kurtosis within ±2, confirming a normal distribution.

**Table 2:** Loadings, Cronbach’s alphas (CA), composite reliability (CR), and average variance extracted (AVE).

Items	Pooled				G1				G2			
	Loadings	CA	CR	AVE	Loadings	CA	CR	AVE	Loadings	CA	CR	AVE
CPEC		0.824	0.892	0.668		0.819	0.889	0.663		0.829	0.895	0.673
CPEC1	0.912				0.909				0.914			
CPEC2	0.920				0.917				0.923			
CPEC3	0.912				0.909				0.917			
CPEC4	0.051*				0.032*				0.073*			
CPEC5	0.910				0.907				0.912			
PDI		0.778	0.862	0.627		0.760	0.851	0.614		0.796	0.872	0.647
PDI1	0.877				0.861				0.893			
PDI2	0.844				0.843				0.845			
PDI3	0.913				0.903				0.926			
PDI4	−0.122*				−0.159*				−0.088*			
PDI5	0.898				0.881				0.915			
UAI		0.752	0.841	0.558		0.760	0.846	0.553		0.743	0.833	0.567
UAI1	0.836				0.804				0.867			
UAI2	0.077*				0.207*				0.069*			
UAI3	0.837				0.827				0.847			
UAI4	0.860				0.862				0.851			
UAI5	0.804				0.806				0.796			
IDV		0.861	0.900	0.645		0.852	0.894	0.630		0.871	0.906	0.662
IDV1	0.835				0.816				0.854			
IDV2	0.878				0.859				0.899			
IDV3	0.875				0.871				0.879			
IDV4	0.698				0.703				0.694			
IDV5	0.711				0.703				0.719			
MAS		0.895	0.923	0.706		0.889	0.919	0.694		0.902	0.927	0.719
MAS1	0.864				0.864				0.863			
MAS2	0.830				0.815				0.846			
MAS3	0.881				0.877				0.886			
MAS4	0.856				0.849				0.864			
MAS5	0.765				0.753				0.777			
L/STO		0.883	0.916	0.686		0.879	0.913	0.679		0.887	0.918	0.694
L/STO1	0.748				0.741				0.754			
L/STO2	0.888				0.887				0.888			
L/STO3	0.874				0.864				0.885			
L/STO4	0.897				0.894				0.900			
L/STO5	0.718				0.714				0.722			

*Items: CPEC4, PDI4, and UAI2 have been removed due to high collinearity between certain items within both constructs. Source: Created by the author from survey data through R-Studio.

**Table 3:** Discriminant validity.

	CPEC	PDI	UAI	IDV	MAS	L/STO
Pooled						
CPEC						
PDI	0.563					
UAI	0.375	0.230				
IDV	0.385	0.259	0.277			
MAS	0.412	0.328	0.398	0.314		
L/STO	0.630	0.581	0.357	0.353	0.738	
Mean	3.967	3.743	3.931	3.887	3.720	3.904
Median	0.921	0.886	0.929	0.924	0.932	0.810
Kurtosis	2.017	1.790	1.160	1.501	0.555	1.010
Skewness	−1.279	−1.162	−1.033	−1.125	−0.888	−0.834
Groups	G1 ***(G2)***					
CPEC						
PDI	0.537 ***(0.590)***					
UAI	0.394 ***(0.357)***	0.305 ***(0.157)***				
IDV	0.378 ***(0.392)***	0.270 ***(0.247)***	0.337 ***(0.214)***			
MAS	0.402 ***(0.422)***	0.337 ***(0.318)***	0.389 ***(0.408)***	0.312 ***(0.316)***		
L/STO	0.607 ***(0.654)***	0.582 ***(0.580)***	0.366 ***(0.348)***	0.358 ***(0.347)***	0.717 ***(0.759)***	
Mean	3.958 ***(3.971)***	3.929 ***(3.888)***	3.912 ***(3.903)***	3.724 ***(3.734)***	3.922 ***(3.928)***	3.752 ***(3.741)***
St.D	0.916 ***(0.921)***	0.912 ***(0.922)***	0.799 ***(0.805)***	0.910 ***(0.925)***	0.868 ***(0.885)***	0.921 ***(0.943)***
Kurtosis	2.052 ***(2.058)***	1.619 ***(1.534)***	1.194 ***(1.081)***	0.631 ***(0.606)***	2.024 ***(1.860)***	1.144 ***(1.074)***
Skewness	−1.281 ***(1.287)***	−1.142 ***(1.125)***	−0.875 ***(−0.838)***	−0.899 ***(0.891)***	−1.195 ***(−1.176)***	−1.009 ***(1.015)***

G1 = Group1 = Non-CPEC regions, G2 = Group2 = CPEC regions. The bold and italic values are G2, and St.D = Standard Deviation. Source: Created by the author from survey data via Smart-PLS.

### 4.3 Structural model assessment

The model fit results demonstrate a strong alignment between the hypothesized and observed data. The explanatory power (R^2^) was 44.2% for pooled data, 40.7% for G1, and 48.5% for G2 (Hair et al., 2023), as shown in Table 4. An SRMR value of 0.048, below the 0.08 threshold, indicates an excellent model fit. Additional indices, such as d_ULS (0.764), d_G (0.637), NFI (0.924), and Chi-square (1358.475), further confirmed the model’s fitness, showing that it is well calibrated and effective in capturing the studied variables’ dynamics.

**Table 4:** Structural model results.

Hypothesis	Relationships			Path coefficient		Path coefficient differences	*P*-value differences (one-tailed)		
		Pooled	Supported	G1	G2	G1-G2	Henseler’s multi-group analysis	Permutation test	Significance
H1	PDI - > CPEC	0.283	Yes	0.255	0.314	−0.059	0.029	0.021	S
H2	UAI - > CPEC	0.141	Yes	0.139	0.158	−0.019	0.216	0.143	Ns
H3	IDV - > CPEC	0.147	Yes	0.135	0.161	−0.026	0.039	0.028	S
H4	MAS - > CPEC	−0.015	No	0.007	−0.040	0.047	0.493	0.376	Ns
H5	L/STO - > CPEC	0.346	Yes	0.330	0.359	−0.030	0.041	0.013	S
R-square		R^2^		R^2^	R^2^				
		44.2%		40.%	48.5%				

G1 = Group1 = Non-CPEC regions; G2 = Group2 = CPEC regions. Source: Created by the author from survey data using Smart-PLS.

### 4.4 Structural model

**Figure 1:**
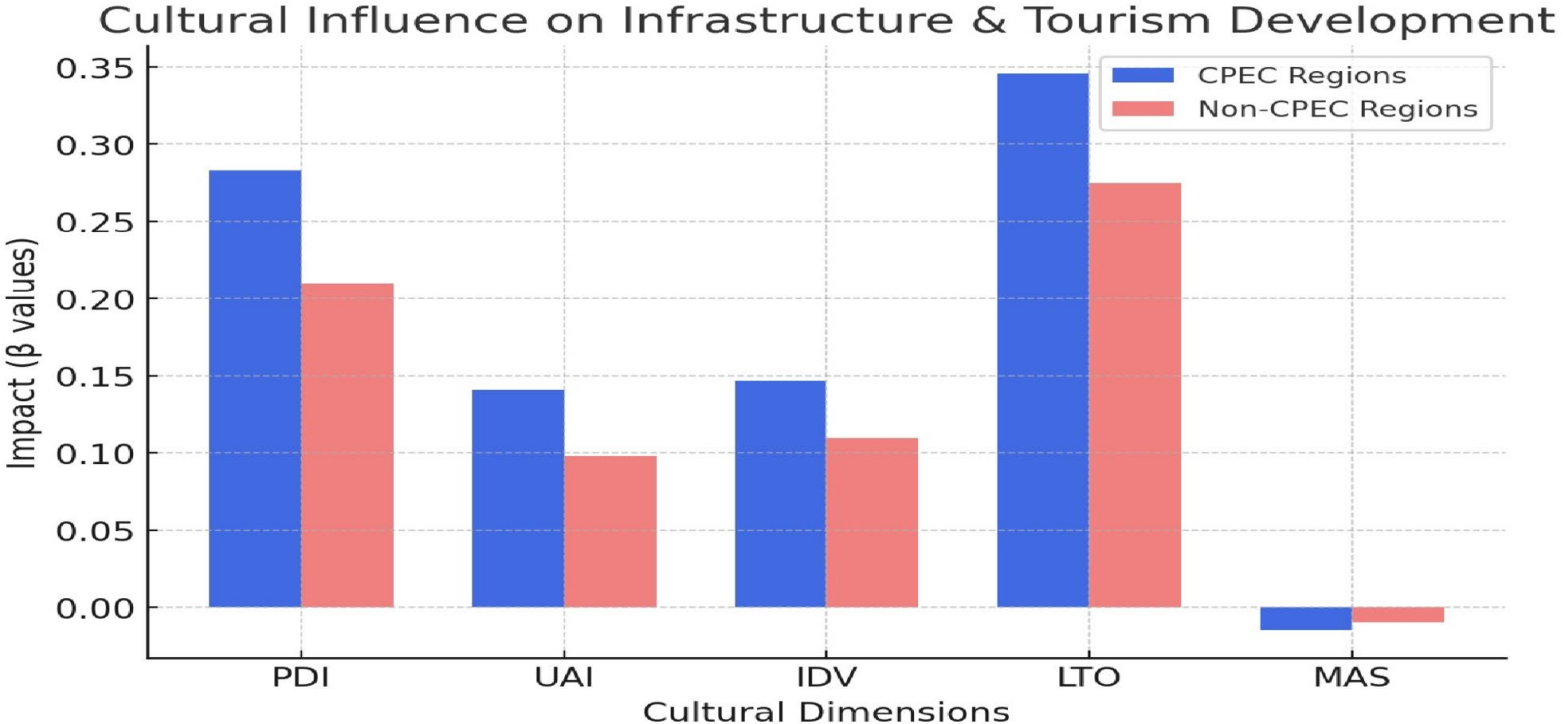
Cultural dimensions influence infrastructure and tourism development. Source: visualized by the author from the PLS-MGA results using RStudio.

The path coefficients were analyzed using PLS-MGA for both G1 and G2 by applying Smart-PLS 4 (Sufriadi et al., 2023), as shown in Table 4 and Figure 1. The structural model results provide significant insights into the relationship between cultural dimensions and CPEC-driven cultural tourism development. As hypothesized, PDI exhibits a strong positive effect on CPEC (*β* = 0.283, *p* < 0.001), supporting H1. This result indicates that regions with higher hierarchical values are more likely to foster cultural tourism. This could be attributed to the centralization of power that typically characterizes regions with high PDI, where authority-motivated environments create stable conditions for tourism development. Similarly, UAI shows a positive relationship (β = 0.141, *p* < 0.001), further supporting H2, indicating that regions prioritizing stability and risk aversion are more favorable to cultural tourism. IDV also positively impacted CPEC (β = 0.147, *p* < 0.001), supports H3, and suggests that individualistic values, including innovation, personal achievement, and local entrepreneurship, contribute to vibrant tourism activities. However, MAS shows a negative coefficient, and CPEC (β = −0.015, *p* = 0.411) does not support H4, highlighting that competitive and achievement-oriented values play a minimal role in cultural tourism development. Notably, L/STO has the strongest positive effect (β = 0.346, *p* < 0.001), thus significantly supporting H5. This finding underscores the importance of future planning and sustainability of CPEC in tourism development. Long-term planning is consistent with the aims of CPEC, which involve infrastructure development and long-term economic and social impacts, making this dimension particularly influential.

### 4.5 Multi-group analysis (MGA)

**Figure 2:**
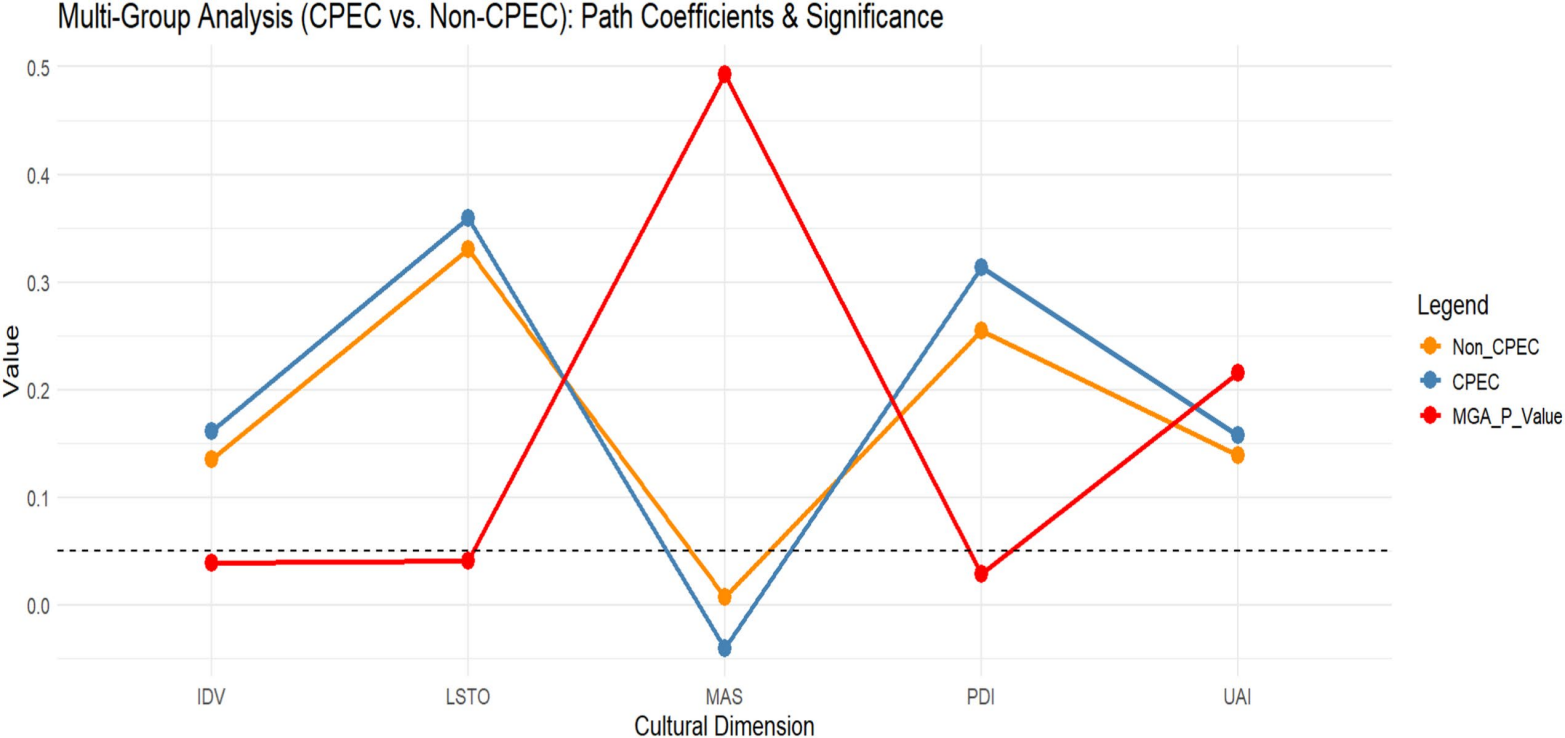
Multi-group analysis. Source: visualized by the author from the multi-group results using RStudio.

The coefficients were analyzed using PLS-MGA for both G1 and G2, as described by Henseler et al. (2016). MGA was conducted to compare the model differences between the two groups, as shown in Table 5. This finding indicates that the PDI effect is stronger in G2 (β = 0.314) than in G1 (β = 0.255), demonstrating that hierarchical values have a more distinct impact on CPEC in G2. The UAI impacts in G2 (β = 0.158) are higher than those in G1 (β = 0.139), indicating that regions prioritizing stability and risk aversion are more attractive to tourists. Similarly, IDV also confirms that G2 (β = 0.161) has a stronger influence on cultural tourism than G1 (β = 0.135). MAS slightly favors G1 (β = 0.007) compared to G2 (−0.040), reflecting that CPEC initiatives did not significantly affect cultural tourism due to its competitive nature. L/STO underscores a stronger effect in G2 (β = 0.359) than G1 (β = 0.330), suggesting that long-term planning is more critical for tourism development in G2. These differences may stem from socioeconomic variations between the groups. Generally, the structural model underscores the importance of cultural dimensions such as PDI, UAI, IDV, and L/STO in shaping CPEC, while also underscoring regional differences in their effects (see Figure 2).

**Table 5:** MICOM results.

C	C==I	CI	PMIE	Difference	CI	Equal	Difference	CI	Equal	Fuzzy Multivariate Interaction Effect
CPEC	1.000	[1.000, 1.000]	Yes	0.018	[−0.124, 0.114]	Yes	0.003	[−0.246, 0.247]	Yes	Yes
PDI	1.000	[1.000, 1.000]	Yes	−0.033	[−0.113, 0.119]	Yes	0.039	[−0.244, 0.243]	Yes	Yes
UAI	0.991	[0.995, 0.986]	Yes	−0.061	[−0.119, 0.124]	Yes	0.179	[−0.210, 0.223]	No	No
IDV	1.000	[0.996, 1.000]	Yes	−0.016	[−0.117, 0.124]	Yes	0.006	[−0.241, 0.245]	Yes	Yes
MAS	1.000	[0.999, 1.000]	Yes	−0.009	[−0.115, 0.112]	Yes	0.057	[−0.189, 0.183]	Yes	Yes
L/STO	1.000	[0.999, 1.000]	Yes	−0.024	[−0.116, 0.115]	Yes	0.031	[−0.236, 0.227]	Yes	Yes

Source: created by the author from survey data using smart-PLS.

### 4.6 MICOM results

The Measurement Invariance of Composite Models (MICOM) was tested following Henseler et al. (2016) and involved three steps: configural invariance, compositional invariance, and equality of means and variances. Full measurement invariance was partially achieved, with configural and compositional invariances fully established across the G1 and G2 regions. However, the equality of means and variances was only partially established initially because the construct UAI failed to meet the criteria for equal variances, as shown in Table 5. Path coefficients were analyzed using PLS-MGA and permutations (Hair et al., 2023). Significant differences favoring Group 2 were observed in the relationships between PDI and CPEC (|Δβ| = 0.059, *p* < 0.05), IDV and CPEC (|Δβ| = 0.026, *p* < 0.05), and L/STO and CPEC (|Δβ| = 0.030, *p* < 0.05). However, no significant differences were found for the UAI (|Δβ| = 0.019, *p* > 0.05) or MAS (|Δβ| = 0.047, *p* > 0.05). This underscores that, while the overall structure of the model is consistent across groups, there are notable differences in how UAI is perceived and operationalized in G1 and G2. For other dimensions, such as PDI, IDV, MAS, and L/STO, full measurement invariance was achieved, demonstrating that these cultural dimensions are interpreted similarly across groups. The results highlight the importance of confirming measurement invariance in multi-group analyses, as it validates the comparability of results across different regions.

### 4.7 Spatial autocorrelation analysis

#### 4.7.1 Global Moran’s I

**Figure 3:**
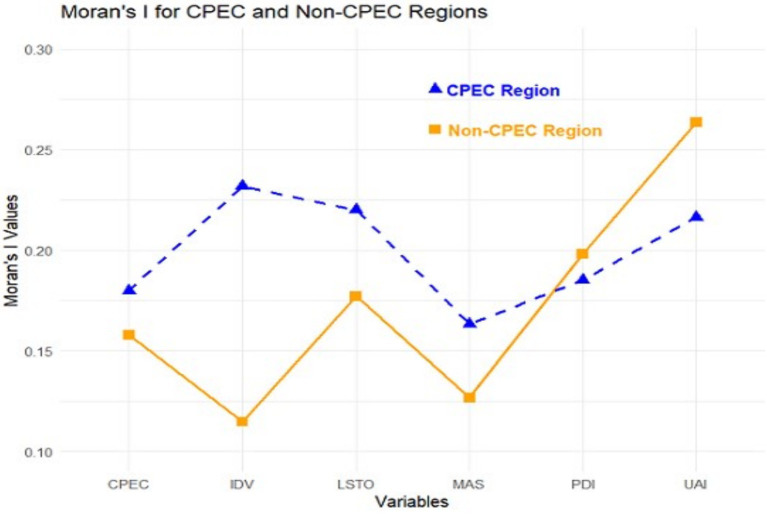
Moran’s I autocorrelation between CPEC and non-CPEC regions. Source: created by the author from survey data using RStudio.

The Global Moran’s I test indicates significant spatial autocorrelation in cultural tourism factors across both CPEC and non-CPEC regions, highlighting a non-random spatial distribution. As shown in Figure 3, both regions exhibit positive spatial autocorrelation, with the majority of variables showing significance at the 0.001 level, whereas the two variables in the non-CPEC region are significant at 0.05. Moran’s I values reflect stronger spatial clustering in CPEC regions compared to non-CPEC regions. For example, Moran’s I = 0.1798 (CPEC regions) and 0.1580 (non-CPEC regions), *p* < 0.001. PDI and UAI variables display high spatial correlations (0.185 and 0.216 in CPEC regions; 0.198 and 0.263 in non-CPEC regions, *p* < 0.001). The IDV and MAS variables also demonstrate significant positive spatial correlations, although they were weaker in the non-CPEC regions (CPEC regions: 0.232, 0.163; non-CPEC regions: 0.115, 0.127, *p* = 0.041–0.001). Finally, the L/STO variable shows a strong positive spatial association (0.220 in the CPEC regions and 0.177 in the non-CPEC regions, both with a *p*-value < 0.001). These findings collectively reveal the spatial association of cultural tourism across both regions, exhibiting a W-shaped upward trend.

#### 4.7.2 Local spatial autocorrelation analysis (LISA)

**Figure 4:**
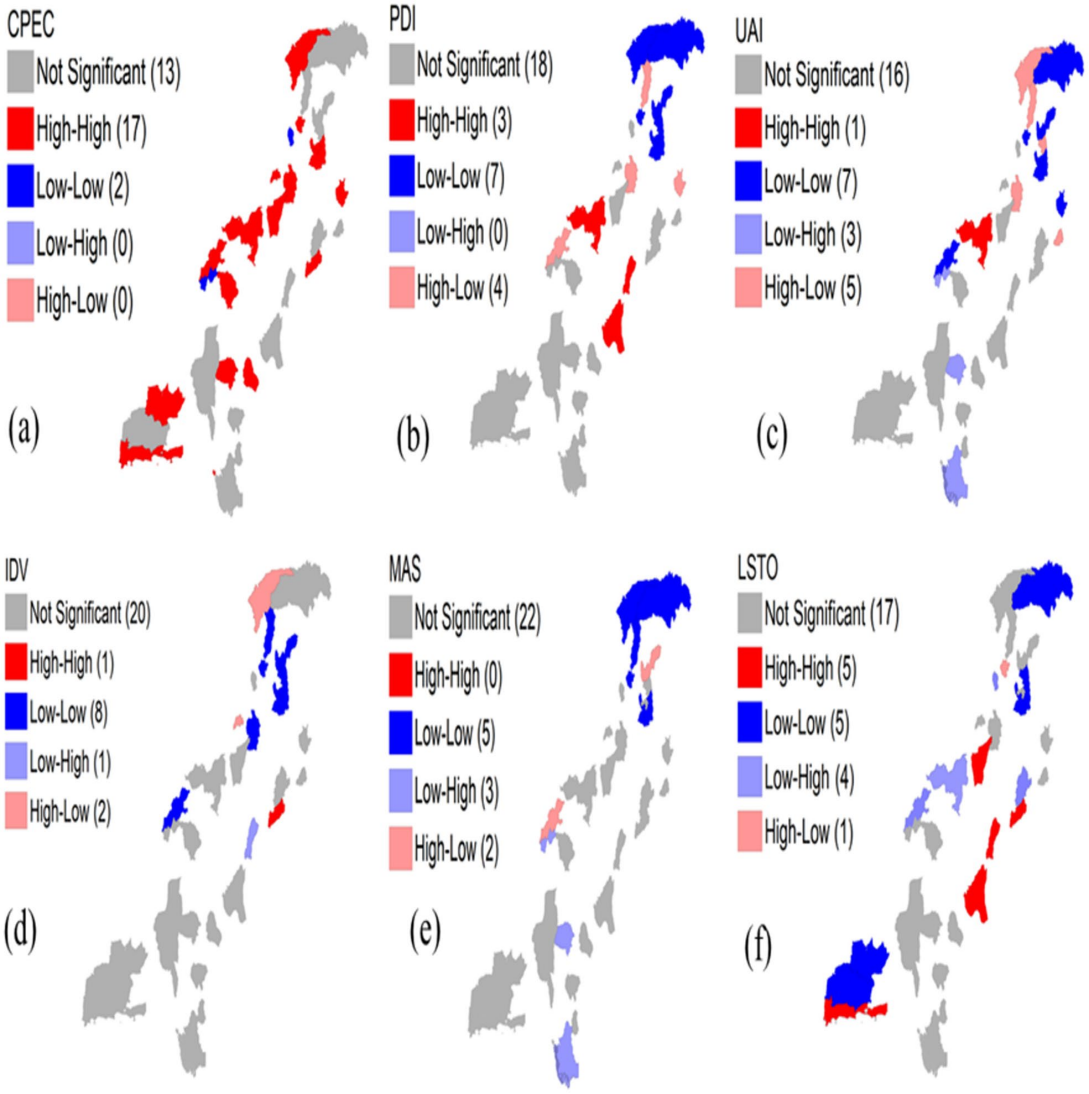
LISA clusters in the CPEC regions. Source: created by the author from survey data visualized using ArcMap and Geoda.

**Figure 5:**
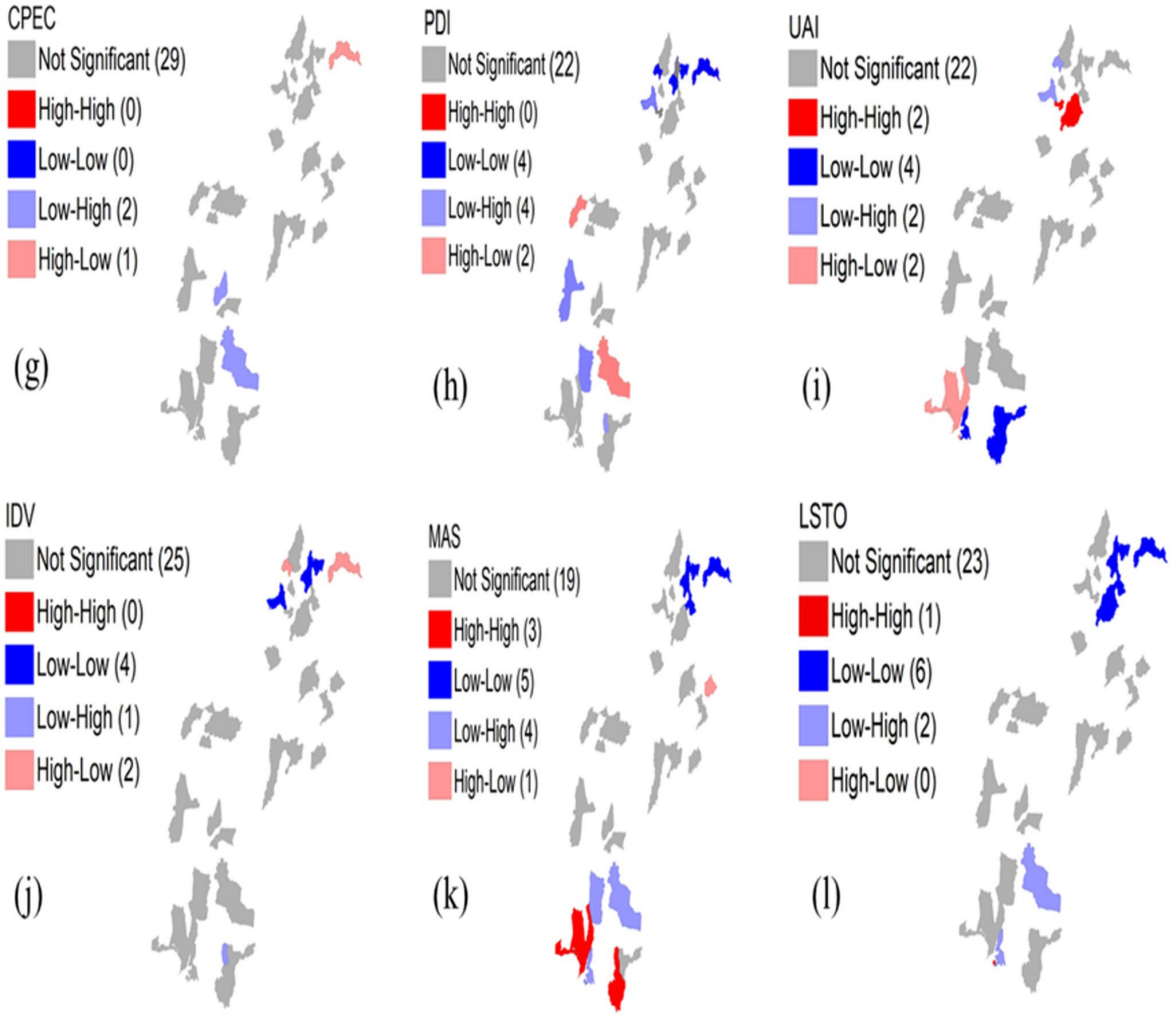
LISA clusters in non-CPEC regions. Source: created by the author from survey data visualized using ArcMap and Geoda.

To further investigate the localized spatial correlation of cultural tourism development in both regions, we employed LISA cluster maps. The LISA cluster analysis for the CPEC regions is shown in Figure 4, whereas the LISA cluster analysis for the non-CPEC regions is shown in Figure 5. These maps reveal distinct patterns between the non-CPEC and CPEC regions. *PDI*: The number of nonsignificant districts decreased from 22 in the non-CPEC region to 18 in the CPEC region. HH clusters emerged in three districts in the CPEC regions from zero in non-CPEC regions, while LL clusters increased from four to seven. LH clusters disappeared in the CPEC regions, reflecting reduced low-PDI districts surrounded by high-PDI areas, whereas HL clusters remained constant in the two districts in both regions. *UAI:* Nonsignificant districts declined from 22 to 16 in the CPEC region. HH clusters reduced slightly, while LL clusters rose from four to seven districts, with LH and HL clusters increasing to three and five districts, respectively, indicating evolving spatial patterns. *IDV:* Nonsignificant districts decreased from 25 to 20 in the CPEC regions. HH clusters emerged in one district of the CPEC region, with LL clusters increasing from four to eight. LH and HL clusters remained stable across both regions.

*MAS:* Nonsignificant districts rose from 19 to 22, with HH clusters decreasing from three to zero in the CPEC regions. This reflected a more diffuse spatial pattern. LL clusters are stable at five, LH clusters slightly decreased, and HL clusters remained consistent at two. *L/STO:* Nonsignificant districts declined from 23 to 17 in the CPEC regions. HH clusters increased from one to five, LL slightly decreased from six to five, LH clusters fell from four to two, and one HL cluster emerged in the CPEC regions, highlighting emerging high L/STO districts within low L/STO regions.

### 4.8 Spatial effects analysis

#### 4.8.1 Selection of spatial econometric model

The presence of significant spatial dependence necessitated spatial modeling. To identify the most appropriate model, LM tests were conducted to assess spatial dependence. Both the LM-lag and LM-error tests, including their robust versions, were significant at a level of 0.05, confirming the need to account for spatial error and lag effects. SDM was tested for potential simplification using LR and Wald tests, which confirmed at the 0.01 level that the SDM could not be reduced to either the SLM or SEM. Therefore, the SDM was selected as the final model.

#### 4.8.2 Model estimation

##### 4.8.2.1 Spatial Durbin model (SDM)

The SDM results provide a nuanced understanding of how cultural dimensions influence tourism development in non-CPEC and CPEC regions (Table 6). In non-CPEC regions, the negative spatial autoregressive coefficient (*ρ* = −1.420, *p* = 0.005) indicates a competitive dynamic where cultural tourism development in one district obstructs neighboring districts. This indicates that resources and tourist attention may be concentrated in specific areas, creating disparities. Conversely, in the CPEC regions, the positive coefficient (ρ = 1.239, *p* = 0.001) suggests a collaborative dynamic, where tourism growth in one district spills over to the adjacent areas. Cultural dimensions also exhibit regional variations. PDI positively influences tourism in both regions (non-CPEC regions: *β* = 0.154, *p* < 0.05; CPEC regions: β = 0.443, *p* < 0.0001), highlighting the role of hierarchical structures in preserving cultural experiences. UAI has a strong positive effect (non-CPEC regions: β = 0.624; CPEC regions: β = 1.303, *p* < 0.001), suggesting a stability-driven tourist attraction. Notably, L/STO shifts from negative in non-CPEC (β = −0.636, *p* < 0.05) to positive in CPEC (β = 0.298, *p* < 0.001), reinforcing long-term planning’s role in tourism sustainability. Similarly, MAS, previously insignificant in non-CPEC regions (β = 0.107, *p* < 0.20), becomes significant in CPEC regions (β = 0.226, *p* < 0.04), possibly because of CPEC-driven socioeconomic fluctuations. These logarithmic transformations further support these findings. PDI and UAI maintain strong positive effects in both regions (non-CPEC regions: ln.PDI = 0.724, ln.UAI = 1.468; CPEC regions: ln.PDI = 0.651, ln.UAI = 1.303, *p* < 0.02), while MAS and L/STO remain insignificant in non-CPEC, but L/STO strengthens and becomes significant in CPEC (β = 0.921, *p* < 0.01). The model fit is robust in both regions but performs better in the CPEC regions (Adjusted R^2^ = 0.683 vs. 0.758). Model selection criteria further confirm this (CPEC: Log Likelihood = 14.516, Akaike Information Criterion = −3.033, Bayesian Information Criterion = 16.022 vs. Non-CPEC: Log Likelihood = 10.897, Akaike Information Criterion = 4.206, Bayesian Information Criterion = 23.601). These results highlight the distinct spatial dynamics shaping tourism in CPEC and non-CPEC regions, necessitating region-specific tourism strategies.

**Table 6:** SDM result estimation.

Regions	Non-CPEC regions			CPEC regions		
Variables	Coefficient	Std. error	t-statistics	Coefficient	Std. error	t-statistic
PDI	0.154**	0.085	1.809	0.443***	0.093	4.7729
UAI	0.624***	0.190	3.278	0.436***	0.104	4.192
IDV	0.266***	0.111	2.919	0.337***	0.115	2.927
MAS	0.107	0.103	1.037	0.226**	0.112	3.014
L/STO	−0.636**	0.272	−2.335	0.298***	0.092	3. 220
ln.PDI	0.724***	0.258	2.803	0.651*	0.292	2.230
ln.UAI	1.468***	0.585	2.510	1.303***	0.298	4.371
ln.IDV	0.643**	0.346	1.861	0.201	0.558	0.359
ln.MAS	−0.494	0.452	−1.095	−0.419	0.399	−1.048
ln.L/STO	−1.942**	0.917	−2.112	0.921***	0.381	2.417
ρ	−1.420***			−1.639***		
Adj-R^2^	0.758			0.683		
Log L	10.897			14.516		
Akaike Information Criterion	4.206			−3.033		
Bayesian Information Criterion	23.601			16.022		

*** *p* < 0.01; ** *p* < 0.05; * *p* < 0.1. Source: Created by the author from survey data using RStudio.

### 4.9 Decomposition of spatial effects

The total, direct, and indirect effects of cultural dimensions on tourism vary between non-CPEC and CPEC regions, as shown in Table 7. In non-CPEC regions, PDI (0.372, *p* < 0.01) and UAI (0.876, *p* < 0.01) generate positive spillover effects, with UAI having the strongest impact. IDV (0.990, *p* < 0.05) also contributes positively, although the direct and indirect effects are mixed. MAS (−0.169, *p* < 0.05) reflects a net negative effect driven largely by indirect influences, whereas L/STO (−1.055, *p* < 0.01) has the strongest negative impact, reinforcing that long-term planning deprioritizes immediate tourism growth. Conversely, in CPEC regions, PDI (0.413, *p* < 0.01) and UAI (0.662, *p* < 0.01) remain significant positive contributors, with UAI playing a key role in fostering tourism. IDV (0.204, *p* < 0.01) supports regional tourism despite some indirect variations. MAS (0.077, *p* < 0.01), which was negative in non-CPEC regions, shifts to a positive effect owing to indirect influences. Unlike in non-CPEC regions, L/STO (0.457, *p* < 0.01) plays a positive and significant role in CPEC regions, highlighting that long-term planning strengthens cultural tourism and benefits neighboring districts.

**Table 7:** Decomposition results of the spatial effect.

Regions	Non-CPEC regions			CPEC regions		
Variables	Direct effect	Indirect effect	Total effect	Direct effect	Indirect effect	Total effect
PDI	0.092*	0.280***	0.372***	0.453***	−0.040**	0.413***
UAI	0.559**	0.318***	0.876***	0.360**	0.302***	0.662***
IDV	−0.232***	0.133**	0.990**	0.381***	0.177***	0.204***
MAS	0.183**	−0.352***	−0.169**	−0.332***	0.409**	0.077***
L/STO	−0.520***	−0.535**	−1.055***	0.244***	0.213***	0.457***

*** *p* < 0.01; ** *p* < 0.05; * *p* < 0.1. Source: Created by the author from survey data through RStudio.

### 4.10 Robustness tests

The robustness of the SDM was rigorously evaluated using alternative spatial weight matrices, Moran’s I on residuals, and robust LM tests, as shown in Table 8. Alternative spatial weight matrices maintained the significance of key variables. Moran’s I test revealed no significant spatial autocorrelation in the residuals for either region, and robust LM tests also indicated no significant unmodeled spatial error or lag components, confirming that the SDM effectively addressed spatial dependencies. These results validate the reliability of the SDM analysis.

**Table 8:** Robustness (alternative spatial weight matrices, Moran’s I and LM tests).

Regions	Non-CPEC regions			CPEC regions		
Variables	Coefficient	Std. error	t-statistics	Coefficient	Std. error	t-statistics
PDI	0.205**	0.092	2.239	0.481***	0.095	5.096
UAI	0.564**	0.204	2.688	0.398***	0.098	4.312
IDV	0.288**	0.109	2.089	0.397***	0.125	3.190
MAS	−0.079	0.111	−0.776	0.286***	0.099	4.114
L/STO	−0.497*	0.267	−1.839	0.281**	0.096	2. 972
ln.PDI	0.579**	0.187	2.199	0.558*	0.387	1.709
ln.UAI	0.825	0.605	0.707	0.875*	0.385	1.971
ln.IDV	0.377**	0.286	0.873	0.184*	0.688	1.502
ln.MAS	−0.925 **	0.327	−2.224	−0.419	0.399	−1.048
ln.L/STO	−1.463 ***	0.815	−1.322	0.921***	0.381	2.417
ρ	−1.420**			−1.823***		
Adj-R^2^	0.571			0.683		
Log L	11.688			13.246		
Akaike Information Criterion	2.624			−1.926		
Bayesian Information Criterion	21. 671			18.128		

Test	Statistic (estimate)	*p*-value	Statistic (estimate)	*p*-value
Moran’s I	−0.078	0.531	−0.784	0.693
LM Error (RSerr)	1.379	0.240	1.547	0.214
LM Lag (RSlag)	0.069	0.801	0.605	0.436
Adj.(RSerr)	2.241	0.134	0.964	0.326
Adj.(RSlag)	0.923	0.336	0 0.023	0.879
SARMA	2.305	0.316	1.570	0.456

Used Moran’s I on residuals and LM tests. *** *p* < 0.01; ** *p* < 0.05; * *p* < 0.1. Source: created by the author from survey data using RStudio.

## 5 Discussion, implications, and limitations

### 5.1 Dynamic mechanism

The dynamic mechanism of cultural tourism in the CPEC and non-CPEC regions highlights the differing impacts of cultural dimensions, infrastructure, and socioeconomic factors. In non-CPEC regions, a higher PDI reflects centralized decision-making, limiting community engagement in tourism. This is consistent with Huang and Crotts (2019), who demonstrated negative correlations between PDI and tourist satisfaction. Conversely, the CPEC regions benefit from decentralized governance and economic opportunities, fostering broader engagement, which is consistent with the findings of Correia et al. (2011). This contrast highlights differences in government policy frameworks: CPEC regions have received targeted policy interventions and multi-level governance support (e.g., SEZ policies, infrastructure investment, and cultural preservation programs), while non-CPEC regions operate under more fragmented development strategies with fewer tourism-specific initiatives. Similarly, UAI exhibits a strong positive correlation with growth in both regions as structured developments under CPEC reduce ambiguity and enhance attraction. These results are consistent with Hofstede (2011) and Kumar and Dhir (2020). IDV significantly influences cultural tourism in CPEC regions, where autonomy drives vibrant tourism activities, while non-CPEC regions reflect collectivist tendencies, aligning with findings by Huang and Crotts (2019) and Lim and Ok (2021). Additionally, MAS shows minimal influence, consistent with Kumar and Dhir (2020), as competitive traits in masculine societies do not foster collaboration.

Finally, L/STO strongly impacts CPEC regions, emphasizing strategic, long-term investments, as noted by Nazarian et al. (2017) and Kumar and Dhir (2020), whereas the lack of coordinated development in non-CPEC regions underscores the need for strategic policies to unlock their tourism potential. This again reflects policy asymmetry: CPEC-specific policies prioritize long-term sustainability and economic integration, whereas non-CPEC areas lack organized policy direction, limiting their capacity to leverage cultural assets.

### 5.2 Spatial spillover effect

Global Moran’s I and LISA cluster analyses reveal significant spatial autocorrelation in cultural tourism across CPEC regions and non-CPEC regions. Higher Moran’s I values in CPEC regions indicate a concentrated, strategically driven development pattern, consistent with the findings of Li et al. (2022), whereas non-CPEC regions display a more diffuse and uncoordinated spatial distribution. LISA further identifies high-high (HH) clusters for PDI and L/STO in CPEC regions, highlighting the impact of targeted investments. The spatial econometric model further underscores regional dynamics, which is consistent with Liao and Liang (2024). In non-CPEC regions, a negative spatial autoregressive coefficient suggests a competitive environment where resource constraints hinder tourism spillovers in neighboring districts. However, cultural dimensions, including PDI, UAI, and IDV, positively impact tourism by reinforcing traditional values and community engagement. Conversely, CPEC regions show a positive spatial autoregressive coefficient, indicating collaborative dynamics where tourism in one district benefits neighboring districts. This effect is reinforced by policy-driven socioeconomic transformations in CPEC regions, such as integrated infrastructure plans, inter-district connectivity projects, and federal support for cultural heritage initiatives. Notably, the positive influence of L/STO underscores the importance of long-term planning for sustainable cultural tourism, aligning with CPEC’s long-term strategic planning efforts. These comparative outcomes indicate that government policy plays a mediating role in cultural tourism development. In the CPEC regions, policy consistency and investment have amplified the positive effects of cultural dimensions and infrastructure. In contrast, policy deficiencies in non-CPEC regions have resulted in fragmented outcomes and limited spillover effects. This finding suggests a more inclusive national tourism policy that bridges the developmental divide and promotes cross-regional learning and coordination.

### 5.3 Theoretical and practical implications

This study extends Hofstede’s cultural dimensions and spatial spillover theories by integrating them into the context of large-scale infrastructure development such as CPEC. This demonstrates how dimensions such as PDI, UAI, IDV, MAS, and L/STO are shaped by infrastructural transformations that influence regional tourism behaviors and perceptions. Unlike traditional applications, this study connects these dimensions to cross-border tourism initiatives, emphasizing their relevance in addressing cultural and spatial dynamics. It further validates SST in cultural tourism by highlighting the competitive dynamics in non-CPEC regions and collaborative patterns in CPEC regions, showing that cultural and socioeconomic contexts mediate spatial development. Practically, these findings advocate coordinated regional strategies to promote sustainable cultural tourism. In CPEC regions, policymakers are encouraged to leverage long-term investments for inclusive, culturally aligned tourism initiatives. Tourism managers should adopt culturally sensitive practices based on Hofstede’s dimensions to cater to diverse visitor needs while preserving cultural heritage. For non-CPEC regions, decentralized, community-driven governance and investments in cross-regional collaborations are vital for reducing resource disparities and fostering competitiveness. Advanced spatial modeling tools are recommended to monitor uneven development patterns and guide targeted interventions, ensuring balanced and sustainable tourism growth.

### 5.4 Limitations and future implications

This study offers valuable insights into the SSE and cultural dynamics of tourism in CPEC and non-CPEC regions. However, this study has several limitations. First, its cross-sectional design restricts our understanding of changes over time. Cultural tourism is an evolving process, especially in the context of ongoing infrastructure development. A longitudinal design in future research could better capture how the relationships between cultural dimensions, infrastructure advancement, and tourism evolve. Second, while the sample size is substantial (1,387 respondents across 64 districts), the sample predominantly comprises students, professionals, and local stakeholders such as employees and entrepreneurs. This may limit the diversity of perspectives by underrepresenting groups, such as small business owners outside the tourism sector, casual tourists, or marginalized communities. Future research should employ broader sampling strategies to enhance representativeness and generalizability. Third, the study’s focus on Hofstede’s cultural dimensions provides a structured framework, but it omits critical socioeconomic and environmental factors such as income inequality, governance incentives (taxes and subsidies), and environmental sustainability. These elements are vital for understanding tourism development and should be integrated into future multi-dimensional models. Fourth, while spatial econometric models (SDM) effectively identify statistical patterns and spatial dependencies, they may not fully capture the nuanced social interactions, community narratives, and individual behaviors that influence tourism decisions.

Future studies could adopt mixed-method approaches to bridge this gap. Finally, the findings are context-specific to the CPEC region. Although this enhances local relevance, it limits its broad applicability. Comparative studies of other regional corridors and developing economies are required to assess the transferability of the results. Furthermore, future research should explore potential negative outcomes, such as cultural homogenization or environmental degradation, to provide a more balanced assessment of infrastructure-driven tourism.

## 6 Conclusion

This study explores the dynamic mechanisms and SSE of cultural tourism in the context of CPEC by integrating Hofstede’s cultural dimension theory with SST. Using SEM, spatial econometric analysis, and stakeholder interviews, this research reveals that CPEC-driven infrastructure and socioeconomic integration significantly enhance cultural tourism by improving accessibility, fostering connectivity, and promoting cultural exchange. In CPEC regions, reduced hierarchical decision-making (low PDI) and lower uncertainty (UAI) due to better infrastructure create inclusive and attractive tourism environments. IDV and L/STO dimensions highlight a focus on autonomy, cultural preservation, and sustainable development, aligning with global practices. The SSE illustrates collaborative and concentrated tourism growth in CPEC regions in contrast to the fragmented and competitive patterns in non-CPEC areas, constrained by limited infrastructure and traditional systems. The spatial econometric model confirms that cultural dimensions such as PDI, UAI, and MAS have stronger positive impacts on CPEC regions because of collaborative governance and long-term planning. This study extends Hofstede’s framework to large-scale infrastructure contexts and empirically validates the SSE in cultural tourism. Practically, it provides policymakers with insights into leveraging coordinated strategies to maximize cultural tourism’s socioeconomic benefits. Future research should focus on the sustainability of these patterns and the broader implications of the CPEC for regional tourism development.

## Data Availability

The raw data supporting this study are available from the corresponding author upon reasonable request.

## Appendix

**Table A1:** Measurement scales.

Construct	Items	Measures	Source
Impacts of CPEC infrastructural development	CPEC 1	Improves infrastructure for tourism.	Hussain (2019), and Zulfaqar et al. (2023)
	CPEC 2	Create employment opportunities.	
	CPEC 3	Enhance quality of life and cultural tourism.	
	CPEC4	Local businesses benefit from tourism.	
	CPEC 5	Infrastructure promotes cultural tourism	
Power Distance Index (PDI)	PDI 1	Clear job instructions are crucial.	Dorfman and Howell (1988), Hofstede (2001), Nazarian et al. (2017), and Filimonau et al. (2018)
	PDI 2	Hierarchical positions are respected.	
	PDI 3	Top manager decisions were not to be questioned.	
	PDI 4	Employees are expected to follow procedures.	
	PDI 5	Employees should not contradict supervisors.	
Uncertainty Avoidance Index (UAI)	UAI 1	Religion as Primitive Beliefs	
	UAI 2	Authority needs to deal with subordinates.	
	UAI 3	People prefer structured situations.	
	UAI 4	Uncertainty creates discomfort.	
	UAI 5	Strict laws reduce uncertainty.	
Individualism vs. Collectivism (IDV)	IDV 1	Independence is valued over collaboration.	
	IDV2	Personal goals can be sacrificed for success.	
	IDV 3	Group success is more important than individuals.	
	IDV 4	Personal freedom exceeds collective interests.	
	IDV 5	Managers should promote group loyalty.	
Masculinity vs. Femininity (MAS)	MAS 1	Men are prioritized for professional careers.	
	MAS 2	Families follow unspoken cultural rules.	
	MAS 3	Competition and achievement are necessary.	
	MAS 4	Women are suited for household tasks.	
	MAS 5	Men reason logically; women use intuition.	
Long/Short-Term Orientation (L/STO)	L/STO 1	Societies value both the past and the future.	
	L/STO 2	Future success exceeds the present goal.	
	L/STO 3	Saving for the future is important.	
	L/STO 4	Traveling to learn new cultures.	
	L/STO 5	Traditions hinder rapid societal changes.	

Source: created by the author.
